# Tumor-Induced Osteomalacia: Increased Level of FGF-23 in a Patient with a Phosphaturic Mesenchymal Tumor at the Tibia Expressing Periostin

**DOI:** 10.1155/2014/729387

**Published:** 2014-08-24

**Authors:** Anke H. Hautmann, Josef Schroeder, Peter Wild, Matthias G. Hautmann, Elisabeth Huber, Patrick Hoffstetter, Martin Fleck, Christiane Girlich

**Affiliations:** ^1^Department of Internal Medicine III, Hematology/Internal Oncology, University Hospital of Regensburg, Franz-Josef-Strauß-Allee 11, 93053 Regensburg, Germany; ^2^Institute of Pathology, University Hospital of Regensburg, 93053 Regensburg, Germany; ^3^Institute of Surgical Pathology, University Hospital of Zurich, 8091 Zurich, Switzerland; ^4^Department of Radiotherapy, University Hospital of Regensburg, 93053 Regensburg, Germany; ^5^Institute of Radiology, Asklepios Clinic, 93077 Bad Abbach, Germany; ^6^Department of Internal Medicine I, University Hospital of Regensburg, 93053 Regensburg, Germany; ^7^Department of Rheumatology and Clinical Immunology, Asklepios Clinic, 93077 Bad Abbach, Germany; ^8^Department of General and Geriatric Medicine, Hospital of Barmherzige Brüder, 93049 Regensburg, Germany

## Abstract

In our case, a 45-year-old male patient had multiple fractures accompanied by hypophosphatemia. FGF-23 levels were significantly increased, and total body magnetic resonance imaging (MRI) revealed a tumor mass located at the distal tibia leading to the diagnosis of tumor-induced osteomalacia (TIO). After resection of the tumor, hypophosphatemia and the increased levels of FGF-23 normalized within a few days. Subsequent microscopic examination and immunohistochemical analysis revealed a phosphaturic mesenchymal tumor mixed connective tissue variant (PMTMCT) showing a positive expression of somatostatin receptor 2A (SSTR2A), CD68, and Periostin. Electron microscopy demonstrated a poorly differentiated mesenchymal tumor with a multifocal giant cell component and evidence of neurosecretory-granules. However, the resected margins showed no tumor-free tissue, and therefore a subsequent postoperative radiotherapy was performed. The patient is still in complete remission after 34 months. Tumor resection of PMTMCTs is the therapy of choice. Subsequent radiotherapy in case of incompletely resected tumors can be an important option to avoid recurrence or metastasis even though this occurs rarely. The prognostic value of expression of Periostin has to be evaluated more precisely in a larger series of patients with TIO.

## 1. Introduction

Tumor-induced osteomalacia (TIO) is a rare, acquired paraneoplastic disorder characterized by a renal phosphate leak leading to hypophosphatemia and deranged bone turnover. The typical biochemical pattern of TIO includes normal circulating levels of calcium and parathormone (PTH), normal or low levels of 1.25-dihydroxyvitamin D (1.25-(OH)_2_D), and elevated levels of alkaline phosphatase [1–6]. The renal phosphate leak manifests itself in hypophosphatemia. Typically, patients with TIO complain of progressive musculoskeletal pain and muscle weakness. During the diagnostic procedure fractures in various localizations are found frequently. The cause of TIO are usually small, slowly growing tumors of mesenchymal origin (phosphaturic mesenchymal tumor mixed connective tissue variant (PMTMCT) [5, 7–10].

In TIO most tumors overexpress the protein fibroblast growth factor-23 (FGF-23) inhibiting renal phosphate reabsorption in the proximal tubules and acting as a phosphaturic factor [1, 3, 11]. In these cases, FGFR-23 levels are increased and immunohistochemical analysis of the tumor is positive [1, 3, 11]. The primary transport protein responsible for phosphate reabsorption in the kidney is the type II sodium-phosphate cotransporter (NPT2a) localized in the proximal tubule. High circulating FGF-23 levels produce renal phosphate wasting through the downregulation of NPT2a [2, 3, 9]. Matrix extracellular phosphoglycoprotein (MEPE) [12] and frizzled related protein-4 (FRP-4) have emerged as candidate mediators of the bone-renal pathophysiology as well but are rarely described in literature, so far [11, 12].

Diagnosis of TIO is often extremely difficult since tumors can be too small for detection by conventional radiological methods. High-resolution magnetic resonance imaging (MRI) of the whole body is the currently proposed method of choice to confirm the location of the tumor. F-18 fluorodeoxyglucose positron emission tomography (FDG-PET) is a very sensitive method [9, 10, 13] but also nonspecific. Mesenchymal tumors often express somatostatin receptors [14]; therefore, octreotide scintigraphy is another functional imaging modality. In recent literature, Gallium Dotatate PET has emerged as a virtually ideal investigation to localize tumors causing TIO as well and performed better than F-FDG PET/CT in some studies and seems to be a promising diagnostic tool in patients in whom 111In-octreotide SPECT/CT prior failed to detect a tumor [15, 16]. Further studies with larger patient population are warranted to validate the data.

In rare cases, a venous sampling of FGF-23 is necessary to detect the tumor [9, 17]. For tumors that cannot be located, medical treatment with phosphate supplements and active vitamin D (calcitriol or alpha calcitriol) is a therapeutic option [9].

If the responsible neoplasm is surgically removed, the abnormalities in phosphate wasting and in vitamin D metabolism typically dissolve in a few days.

## 2. Materials and Methods

The described patient has given written consent for publication of the data and the photographs of the histopathology of the tumor. The following antibodies were used for the immunohistochemical analysis performed at the Institute for Surgical Pathology in Zurich: somatostatin receptor 2A (SSTR2A), polyclonal antibody, dilution 1 : 100, Zytomed Systems; OSF-2/Periostin, monoclonal antibody, dilution 1 : 1000, R&D Systems; CD 68, monoclonal antibody, dilution 1 : 50, DAKO A/S. Human FGF-23 c-terminal was measured in the laboratories of Synlab Weiden, Germany. An ELISA kit of the Company, Immutopics, Inc., was used.

## 3. Case Report

A 45-year-old man was admitted to our tertiary Rheumatology center due to acute aggravation of systemic bone pain. The symptoms (pain predominantly in knees, heels, and costosternal joints) worsened gradually during the last weeks but had developed one year previously. The patient had a medical history of hypertension and denied any family history of metabolic bone disease.

Upon physical examination (weight: 96 kg, height: 173 cm, and BMI: 32 kg/m^2^), only painful knees and heels without swelling were conspicuous. Laboratory data are shown in Table 1. A 24-hour urine sample revealed an increased phosphate clearance of 44.7 mL/min (normal range: 5–16 mL/min). Fractional excretion of phosphate was increased with 19.2% (normal range: <5% in the setting of hypophosphatemia). Radiological images revealed beginning degenerative signs. A 700 MBq technetium-Teceos bone scan demonstrated increased uptake in the proximal right tibia and fibula, the right calcaneus, the ventral ribs, and both femoral necks. An MRI of the right knee and right calcaneus showed several months old fracture of the proximal tibia and a capillary fracture of the calcaneus.

The dual energy X-ray absorptiometry (DXA) scan showed a low mineral density (Z-score of the lumbar spine: −1.4; Z-score of the femoral neck: −1.5). A bone biopsy of the proximal tibia revealed no signs for fibrosis, osteomalacia, a neoplasm, or a Morbus Paget.

**Figure 1:**
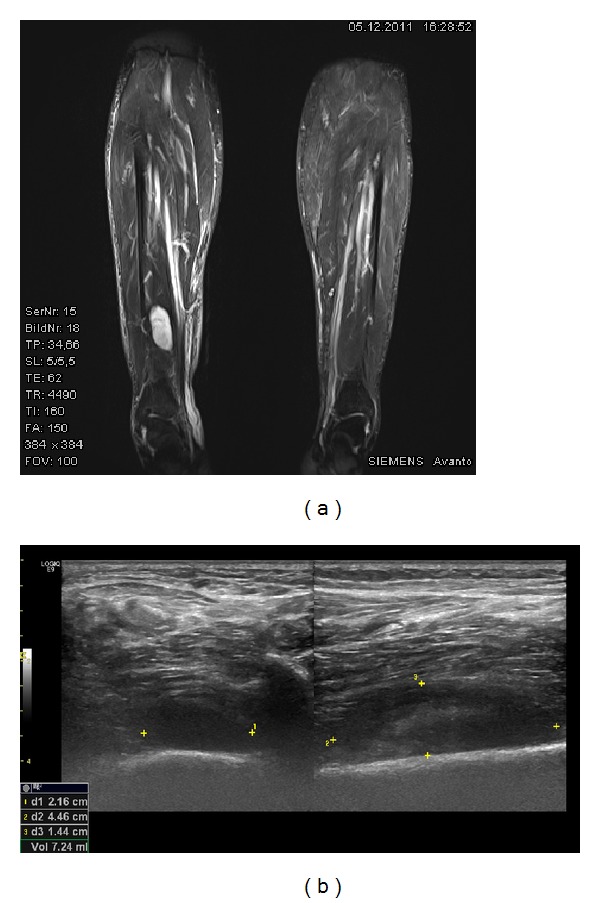
(a) MRI of the giant cell tumor at the distal tibia and (b) an ultrasound examination of the tumor.

Based on the differential diagnosis of a TIO, an MRI of the whole body was performed revealing a tumor, measuring 5.2 × 2.2 × 1.5 cm in size, located at the right dorsal distal tibia. On T1-weighted images the tumor showed isointensity with the muscle tissue, on T2-weighted images hyperintensity (Figure 1(a)). Complementary, an examination with ultrasound was performed showing a tumor of low echogenicity (Figure 1(b)).

The diagnosis of primary TIO was confirmed without evidence of a secondary TIO (PSA in normal range, inconspicuous CT scan of the lung). Consequently, a tumor excision was performed in the Department of Trauma Surgery, University of Regensburg. Before the operation the level of human FGF-23 c-terminal was 0.545 RU/mL (normal range: 0.026–0.110 RU/mL). The level of 1.25-(OH)_2_D was decreased to 2.5 pg/mL despite substitution. The patient still showed hypophosphatemia with a level of 1.83 mg/dL despite oral substitution.

**Figure 2:**
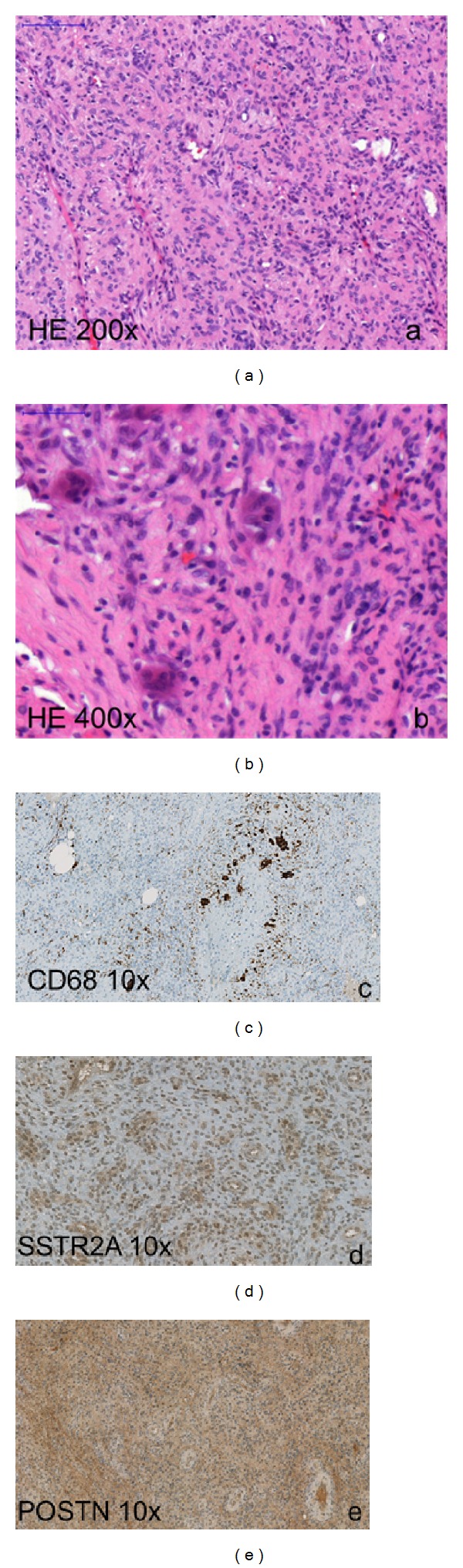
Phosphaturic mesenchymal tumor mixed connective tissue variant. (a) Histopathology of the tumor consisting of monomorphic cells with round cell nuclei without increased mitotic activity (hematoxylin-eosin stain: HE 200x), (b) scattered multinucleated giant cells (HE 400x), and ((c)–(e)) expression of CD68, SSTR2, and POSTN in tumor tissue (immunohistochemistry).

**Figure 3:**
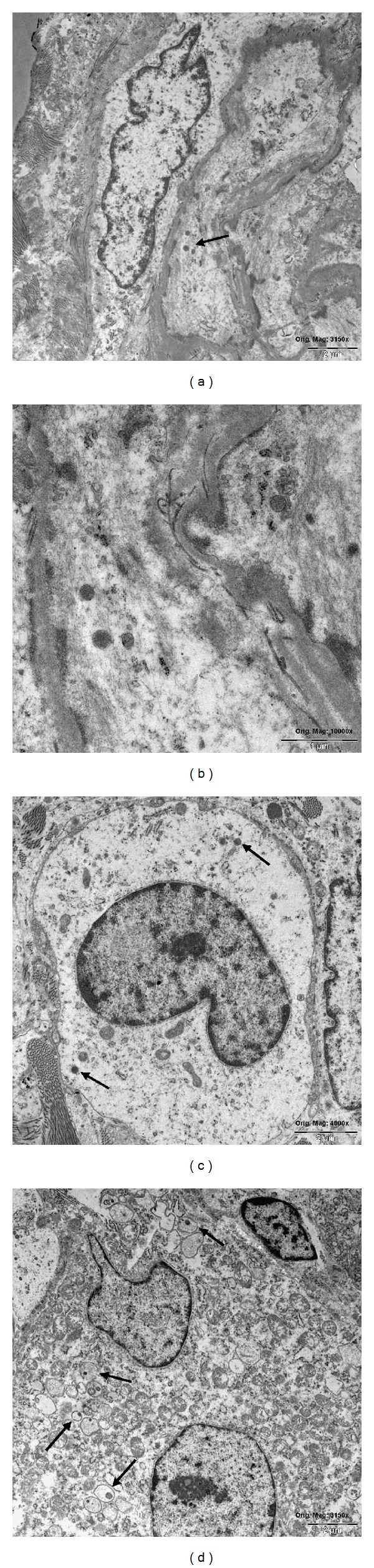
Primary formalin fixed tumor tissue and electron microscopic images. (a) Ultrastructural features of spindle shaped stromal cells with organelle-poor cytoplasm and few dense granules (arrow). Orig. mag. × 3125. (b) The granules resemble neurosecretory granules, diameter approx. 200 nm (not typical “dense core” granules), orig. mag. × 10,000. (c) Rounded tumor cell with a nuclear indentation, scant organella, and any cell surface specialized structures. Note singular electron dense granules in cytoplasm (arrows). Orig. mag. × 4,000. (d) Ultrastructural aspect of a giant multinuclear cell displaying in the organelle-rich cytoplasm some vesicular structures with pericentric dense inclusions (arrows). Orig. mag. × 3125.

The histopathology of the resected tissue revealed a PMTMCT, measuring 5 × 2 × 1.5 cm in size. The tumor consisted of monomorphic cells with round cell nuclei without increased mitotic activity (MIB 1 < 2%) and scattered giant cells in small groups without signs of malignancy (Figures 2(a) and 2(b)). The tumor showed no necrosis and no calcification. The histopathology in context with the clinical findings is suitable for a PMTMC. However, the resected margins showed no tumor-free tissue. The immunohistochemical staining revealed a positive expression of SSTR2A, CD68, and POSTN (OSF-2/Periostin) (Figures 2(c)–2(e)). Complementarily, an electron microscopy investigation was performed revealing an admixture of spindle to pleomorphic shaped cells with deep indentations of the nuclear membrane and scant cell organella in the cytoplasm. The cells displayed some short RER profiles, few mitochondria, dispersed cytoplasmic filaments of intermediate type; in some of them singular inclusions resembling neurosecretory granules were found (Figures 3(a) and 3(b)). These membrane-bound structures had a diameter of approximately 200 nm and sometimes a hardly visible “dense core” could be recognized (primarily formalin fixed material). These cells were densely dispersed in a collagen rich matrix with numerous small blood vessels—most of them without a formed lumen. In the matrix diffuse dispersed mast cells and singular giant cells (some multinuclear) with a histiocytic phenotype were found. Some of the giant cells contained vesicular structures with pericentric located dense granules resembling secretory inclusions similar to those observed in, for example, insulinoma (Figure 3(c)). Singular giant cells showed randomly oriented sarcomeric structures (Figure 3(d)).

Since the tumor was not resected totally, radiotherapy of the right lower extremity has subsequently been performed, as a relapse of a benign giant cell tumor, or PMTMCT has been described [2, 9, 18, 19]. The case was discussed controversially in our interdisciplinary conference of tumors because metastasis in PMTMCTs is rarely described. Because of low toxicity (relatively small clinical target volume (CTV) for irradiation and large space to critical organs of risk) the decision came to perform radiotherapy to avoid the risk of later recurrence or metastasis. In case of recurrence a reresection could most likely have led to distinct loss of function of the limb. Including image fused CT- and MRI-scans for treatment planning, the CTV consisted of the whole compartment including bone structures of the lower extremity. The radiotherapy was performed with a daily dosage of 1.8 Gy (6 and 15 MV photons) five times a week until a cumulative dose of 45 Gy (acute side effects: radiodermatitis CTC grade I).

Two days after resection of the tumor, the level of FGF-23 c-terminal was within the normal range (0.037 RU/mL, normal range: 0.026–0.110 RU/mL) and treatment with phosphate was stopped. The level of alkaline phosphatase was still elevated at 276 U/L. The patient reported a significant improvement of his pain in the knees and heels allowing almost complete reduction of analgesics.

Two months later, laboratory data showed normal levels for phosphate, calcium, and FGF-23 c-terminal (0.054 RU/mL). The alkaline phosphatase was still elevated at 178 U/L; the level of 25-(OH)-D was decreased with 17.3 ng/mL; the level of 1.25-(OH)-D was normal. An MRI performed on the tibia showed no relapse of the giant cell tumor.

There was a follow-up visit 26, 29, and 34 months after establishment of diagnosis. The follow-up data including laboratory results and DXA has been performed again and showed normal levels of FGF-23 c-terminal and vitamin D (see Table 1) and a normal mineral density of bone. The level of phosphate was slightly decreased with 2.14 mg/dL, and an oral substitution was restarted. The level of phosphate turned to normal at last follow-up. An MRI of the tibia showed no relapse. Due to disease remission, additional radiographic investigations were not performed.

## 4. Discussion

This case report presents a patient with a PMTMCT leading to the rare disease TIO.

The histopathology of tumors leading to TIO reveals in 70–80% of cases PMTMCTs [2] which are rare neoplasms occurring in approximately 53% of cases within bones, in 45% in soft tissue and in 3% of patients in the skin. PMTMCT is normally benign, but malignant variants have already been described [2, 19, 20].

Folpe and colleagues [19] performed a series of immunohistochemical staining in tumors leading to TIO. They found a positive staining for FGF-23 in about 70% of all cases; all other markers remained negative. FGF-23 overexpression produced predominantly in bone suppresses osteoblast differentiation as well as matrix mineralization, which suggests that FGF-23 acts directly on bone by reducing mineralization [12]. Furthermore, somatostatin receptors have been found to be present in many TIO tumors [14, 17, 21]. Houang et al. showed in all 15 tumors of 14 patients with TIO a positive staining for FGF-23 and SSTR2A. They concluded that a positive staining for both markers is highly sensitive to PMTs, but not specific. A negative staining can serve as an excellent rule-out test for this diagnosis [21]. In our case, the immunohistochemical staining for SSTR2A, CD 68, and Periostin was positive (Figures 2(c)–2(e)); the staining for FGF-23 could not be established. However, given that FGF-23 serum levels normalized after resection it is clear that the tumor must have produced FGF-23.

To our knowledge a positive staining of Periostin of PMTs of patients with TIO has so far not been described in the literature. Periostin is a newly identified mediator of the inflammatory process and seems to play a role in collagen fibrogenesis and tumor development. It is an extracellular matrix protein expressed by fibroblasts and has been observed in a variety of human malignancies predicting a poor prognosis [22–25]. Several studies revealed that Periostin is upregulated in a wide variety of cancers such as colon, pancreatic, ovarian, breast, non-small cell lung, and head and neck cancer [26, 27]. Periostin binding to the integrins activates the Akt/PKB- and FAK-mediated signaling pathways which lead to increased cell survival, angiogenesis, invasion, and epithelial-mesenchymal transition of carcinoma cells [24, 27]. Furthermore Periostin expression is upregulated and associated with myocardial fibrosis in patients with heart failure and seems to play a role in various inflammatory settings [28]. In patients with non-small cell lung cancer (NSCLC) a positive immunostaining of Periostin in the mesenchymal areas, but not in the cancer cells themselves, could be demonstrated. The patients with tumors exhibiting high-level Periostin expression showed a significantly shorter survival time [22]. The results of Hong et al. showed that the overexpression of Periostin predicts a poor prognosis; therefore the authors conclude that overexpression of Periostin could be regarded as a novel molecule in the progression and development of NSCLC. Further studies have to follow to evaluate the role of Periostin expression in patients with TIO. Periostin upregulation might be an indirect marker for a higher risk of recurrence or development of metastasis. The positive expression of Periostin supports the option of subsequent postoperative radiotherapy in incompletely resected tumors.

In terms of ultrastructural features, neurosecretory granules were found similar to a neuroendocrine tumor [9, 29–31]. However, in these studies immunostaining for typical markers of neurosecretory tumors was negative.

In our case report, the electron microscopy investigation showed ultrastructural features of spindle shaped stromal cells with organelle-poor cytoplasm and a few dense granules resembling neurosecretory granules. These findings are consistent with the majority of the literature [29–31]. However, some PMTs do not show neurosecretory granules [32]. Electron microscopy is a helpful complementary diagnostic tool in ambiguous histopathology.

Late recurrence due to metastatic disease is rare but possible, occurring in less than 5% of the patients with TIO [9, 19, 33]. A small subset of apparently benign giant cell tumors develops hematogenous metastases, usually of the lung [34]. Because of the positive margins of the resected tissue in our patient a subsequent radiotherapy was initiated. The case was discussed controversially in our interdisciplinary conference of tumors because metastasis in PMTMCTs is rarely described. Because of low toxicity (see page 6) the decision came to perform radiotherapy to avoid the risk of later recurrence or metastasis because of incompletely resected margins of the tumor. Furthermore, in case of recurrence a reresection could most likely have led to distinct loss of function of the limb. Further studies are needed in patients with incompletely resected tumors to determine the postoperative treatment options.

Caudell and colleagues [18] performed a retrospective analysis of 25 consecutive patients with giant cell tumors undergoing radiotherapy adjuvant to surgery, or as an alternative treatment in unresectable cases. When radiotherapy is used for primary treatment, the rate of local control seems to be satisfactory, whereas patients treated with radiotherapy for recurrent tumors had worse outcomes [18]. Incompletely resected giant cell tumors or lesions that are surgically inaccessible are usually treated with moderate-dose radiotherapy (45–50 Gy) and have a 65% to 90% likelihood of being locally controlled [35]. No severe acute or chronic toxicity has been reported following radiotherapy [34–37].

In conclusion, TIO is a paraneoplastic syndrome caused by the phosphaturic hormone FGF-23 that can be cured by removing the responsible neoplasm. Subsequent radiotherapy in case of incompletely resected tumors can be an important option to avoid recurrence or metastasis even though this occurs rarely. Especially if a recurrence with the need of re-resection would lead to distinct function of a limb an additive radiotherapy in case of incompletely resected tumors can be recommended. The prognostic value of expression of Periostin has to be evaluated more precisely in a larger series of patients with TIO.

## Figures and Tables

**Table 1:** Laboratory data of the patient before and after resection of the giant cell tumor.

Laboratory value (normal range)	Before resection	2 days after resection	2 months after resection	26 months after resection	29 months after resection	Last follow-up, 34 months after resection
FGF-23 c-terminal (0.026–0.110 RU/mL or 20–70 pg/mL	0.396 (+)	0.037 (normal)	Normal	Normal	Normal	Normal
Phosphate (2.6–4.5 mg/dL)	1.95 (−)	n.p.	Normal	Normal	2.14 (−)	Normal
Calcium (8.5–10.1 mg/dL)	Normal	Normal	Normal	Normal	Normal	Normal
Alkaline phosphatase (40–129 U/L)	305 U/L	276 U/L	178 U/L	Normal	Normal	Normal
Bone specific alkaline phosphatase (<75%)	79.6%	n.p.	n.p.	n.p.	Normal	Normal
1.25-(OH)_2_D (16–81 pg/mL)	4.83 (−)	n.p.	Normal	Normal	Normal	Normal
25-(OH)-D (20–60 ng/mL)	21 (normal)	n.p.	17.3 (−)	Normal	Normal	Normal
Parathyroid hormone (15–65 ng/L)	Normal	Normal	Normal	Normal	Normal	Normal

n.p. = not performed.
